# (2*E*)-1-(5-Chloro­thio­phen-2-yl)-3-(2,3-dimeth­oxy­phen­yl)prop-2-en-1-one

**DOI:** 10.1107/S1600536811037135

**Published:** 2011-09-17

**Authors:** A. N. Prabhu, A. Jayarama, Ravish Sankolli, T. N. Guru Row, V. Upadhyaya

**Affiliations:** a Physics Department, Manipal Institute of Technology, Manipal University, Manipal 576 104, India; b Department of Physics, Mangalore Institute of Technology & Engineering (MITE), Badagamijar, Moodabidri, Karnataka, India; c Solid State and Sructural Chemistry Unit, Indian Institute of Science, Bangalore 560 012, India

## Abstract

In the title compound, C_15_H_13_ClO_3_S, the chloro­thio­phene and dimeth­oxy­phenyl groups are linked by a prop-2-en-1-one group. The C=C double bond exhibits an *E* conformation. The mol­ecule is non-planar, with a dihedral angle of 31.12 (5)° between the chloro­thio­phene and dimeth­oxy­phenyl rings. The meth­oxy group at position 3 is coplanar with the benzene ring to which it is attached, with a C—O—C—C torsion angle of −3.8 (3)°. The meth­oxy group attached at position 2 of the benzene ring is in a (+)synclinal conformation, as indicated by the C—O—C—C torsion angle of −73.6 (2)°. In the crystal, two different C—H⋯O inter­molecular inter­actions generate chains of mol­ecules extending along the *b* axis.

## Related literature

For general background to chalcones and their biological properties, see: Choudary *et al.* (1999 ▶); Tomazela *et al.* (2000 ▶). For a related structure, see: Benmekhbi *et al.* (2009 ▶).
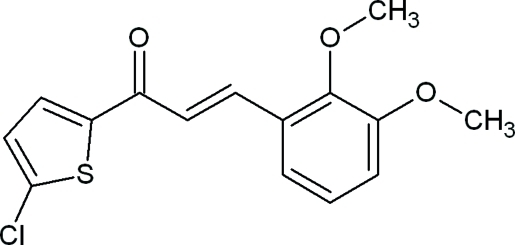


## Experimental

### 

#### Crystal data

C_15_H_13_ClO_3_S
*M* *_r_* = 308.76
Monoclinic, 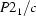

*a* = 11.6139 (8) Å
*b* = 8.5605 (5) Å
*c* = 14.4174 (9) Å
β = 99.907 (2)°
*V* = 1412.02 (16) Å^3^
*Z* = 4
Mo *K*α radiation
μ = 0.42 mm^−1^
*T* = 296 K
0.20 × 0.18 × 0.16 mm

#### Data collection

Bruker SMART APEX CCD detector diffractometer
Absorption correction: multi-scan (*SADABS*; Bruker, 1998 ▶) *T*_min_ = 0.920, *T*_max_ = 0.936
9286 measured reflections
3087 independent reflections
2348 reflections with *I* > 2σ(*I*)
*R*_int_ = 0.026

#### Refinement

*R*[*F* ^2^ > 2σ(*F* ^2^)] = 0.038
*wR*(*F* ^2^) = 0.102
*S* = 1.07
3087 reflections
183 parameters
H-atom parameters constrained
Δρ_max_ = 0.21 e Å^−3^
Δρ_min_ = −0.26 e Å^−3^

### 

Data collection: *SMART* (Bruker, 1998 ▶); cell refinement: *SAINT-Plus* (Bruker, 1998 ▶); data reduction: *SAINT-Plus*; program(s) used to solve structure: *SHELXS97* (Sheldrick, 2008 ▶); program(s) used to refine structure: *SHELXL97* (Sheldrick, 2008 ▶); molecular graphics: *ORTEP-3* (Farrugia, 1997 ▶) and *CAMERON* (Watkin *et al.*, 1996 ▶); software used to prepare material for publication: *WinGX* (Farrugia, 1999 ▶).

## Supplementary Material

Crystal structure: contains datablock(s) global, I. DOI: 10.1107/S1600536811037135/pv2439sup1.cif

Structure factors: contains datablock(s) I. DOI: 10.1107/S1600536811037135/pv2439Isup2.hkl

Supplementary material file. DOI: 10.1107/S1600536811037135/pv2439Isup3.cml

Additional supplementary materials:  crystallographic information; 3D view; checkCIF report

## Figures and Tables

**Table 1:** Hydrogen-bond geometry (Å, °)

*D*—H⋯*A*	*D*—H	H⋯*A*	*D*⋯*A*	*D*—H⋯*A*
C2—H2⋯O1^i^	0.93	2.53	3.209 (2)	130
C15—H15*A*⋯O2^ii^	0.96	2.55	3.423 (3)	151

Symmetry codes: (i) 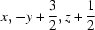; (ii) 
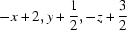
.

**Fig. 1.**
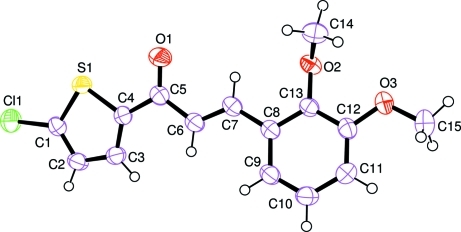
ORTEP (Farrugia, 1997) view of the title compound, showing 50% probability ellipsoids and the atom numbering scheme.

**Fig. 2.**
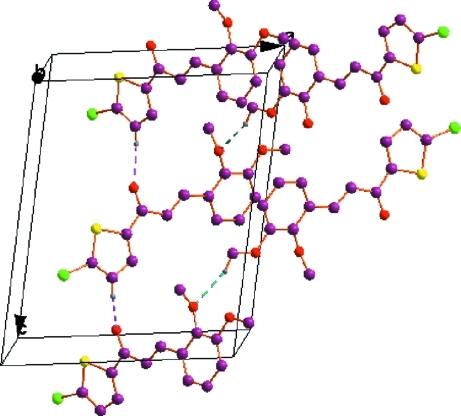
A unit cell packing of the title compound showing intermolecular interactions with dotted lines. H-atoms not involved in hydrogen bonding have been excluded for clarity.

## supplementary crystallographic information

### Comment

Chalcones represent one of the most abundant and ubiquitous group of natural products (Tomazela, *et al.*, 2000). In the past few years, they have been shown to possess interesting biological properties and synthetic intermediates (Choudary, *et al.*, 1999). In the title compound (Fig. 1), the chlorothiophene and dimethoxyphenyl groups are linked by a prop-2-en-1-one group. The chlorothiophene and dimethoxyphenyl rings are non-planar with the dihedral angle 31.12 (5)°. The torsion angle C15–O3–C12–C11 is 3.7 (3)° indicating that the O3 methoxy group is coplanar with the attached benzene ring. The other methoxy group at O2 is in a + synclinal conformation as indicated by the torsion angle 73.6 (2)° for C14–O2–C13–C12. The C═C double bond exhibits an E conformation (Benmekhbi, *et al.*, 2009). The crystal structure is stabilized by two different C—H···O intermolecular interactions generating chains of molecules along the *b*-axis (Fig. 2).

### Experimental

To synthesizethe the title compound, 2-acetyl-5-chlorothiophene (0.01 mol) and 2,3-dimethoxybenzaldehyde (0.01 mol) were dissolved in methanol (60 ml). Sodium hydroxide (5 ml, 20%) was then added drop wise to the solution, and it was stirred for 2 h. The contents of the flask were poured into ice-cold water, and the resulting crude solid was collected by filtration. The compound was dried and re-crystallized twice from acetone.

### Refinement

The H atoms were placed at calculated positions in the riding model approximation with C—H = 0.93 and 0.96 Å, for aryl and methyl H-atoms, respectively, with *U*_iso_(H) = 1.2 and 1.5*U*_eq_(C).

### Crystal data

**Table:**

C_15_H_13_ClO_3_S	*F*(000) = 640
*M**_r_* = 308.76	*D*_x_ = 1.452 Mg m^−^^3^
Monoclinic, *P*2_1_/*c*	Mo *K*α radiation, λ = 0.71073 Å
Hall symbol: -P 2ybc	Cell parameters from 3087 reflections
*a* = 11.6139 (8) Å	θ = 1.8–27.0°
*b* = 8.5605 (5) Å	µ = 0.42 mm^−^^1^
*c* = 14.4174 (9) Å	*T* = 296 K
β = 99.907 (2)°	Block, yellow
*V* = 1412.02 (16) Å^3^	0.20 × 0.18 × 0.16 mm
*Z* = 4	

### Data collection

**Table:**

Bruker SMART APEX CCD detector diffractometer	3087 independent reflections
Radiation source: fine-focus sealed tube	2348 reflections with *I* > 2σ(*I*)
graphite	*R*_int_ = 0.026
ω scans	θ_max_ = 27.0°, θ_min_ = 1.8°
Absorption correction: multi-scan (*SADABS*; Bruker, 1998)	*h* = −14→14
*T*_min_ = 0.920, *T*_max_ = 0.936	*k* = −8→10
9286 measured reflections	*l* = −18→14

### Refinement

**Table:**

Refinement on *F*^2^	Primary atom site location: structure-invariant direct methods
Least-squares matrix: full	Secondary atom site location: difference Fourier map
*R*[*F*^2^ > 2σ(*F*^2^)] = 0.038	Hydrogen site location: inferred from neighbouring sites
*wR*(*F*^2^) = 0.102	H-atom parameters constrained
*S* = 1.07	*w* = 1/[σ^2^(*F*_o_^2^) + (0.0494*P*)^2^ + 0.1854*P*] where *P* = (*F*_o_^2^ + 2*F*_c_^2^)/3
3087 reflections	(Δ/σ)_max_ = 0.001
183 parameters	Δρ_max_ = 0.21 e Å^−^^3^
0 restraints	Δρ_min_ = −0.26 e Å^−^^3^

### Special details

**Table:**

Geometry. All s.u.'s (except the s.u. in the dihedral angle between two l.s. planes) are estimated using the full covariance matrix. The cell s.u.'s are taken into account individually in the estimation of s.u.'s in distances, angles and torsion angles; correlations between s.u.'s in cell parameters are only used when they are defined by crystal symmetry. An approximate (isotropic) treatment of cell s.u.'s is used for estimating s.u.'s involving l.s. planes.
Refinement. Refinement of *F*^2^ against ALL reflections. The weighted *R*-factor *wR* and goodness of fit *S* are based on *F*^2^, conventional *R*-factors *R* are based on *F*, with *F* set to zero for negative *F*^2^. The threshold expression of *F*^2^ > 2σ(*F*^2^) is used only for calculating *R*-factors(gt) *etc*. and is not relevant to the choice of reflections for refinement. *R*-factors based on *F*^2^ are statistically about twice as large as those based on *F*, and *R*- factors based on ALL data will be even larger.

### Fractional atomic coordinates and isotropic or equivalent isotropic displacement parameters (Å^2^)

**Table:**

	*x*	*y*	*z*	*U*_iso_*/*U*_eq_	
Cl1	0.22862 (5)	0.40719 (7)	1.17836 (4)	0.06157 (19)	
S1	0.33583 (4)	0.57846 (6)	1.03539 (3)	0.04070 (15)	
O1	0.46583 (12)	0.75106 (18)	0.91191 (10)	0.0540 (4)	
O2	0.80027 (11)	0.99907 (15)	0.81682 (8)	0.0413 (3)	
O3	0.97557 (11)	1.20940 (17)	0.83929 (10)	0.0509 (4)	
C1	0.33815 (16)	0.5228 (2)	1.14971 (13)	0.0387 (4)	
C2	0.43004 (17)	0.5799 (2)	1.21037 (13)	0.0451 (5)	
H2	0.4440	0.5608	1.2748	0.054*	
C3	0.50212 (16)	0.6722 (2)	1.16311 (13)	0.0427 (5)	
H3	0.5697	0.7210	1.1936	0.051*	
C4	0.46355 (14)	0.6834 (2)	1.06827 (12)	0.0358 (4)	
C5	0.51284 (15)	0.7641 (2)	0.99432 (13)	0.0384 (4)	
C6	0.61819 (15)	0.8604 (2)	1.02313 (13)	0.0407 (4)	
H6	0.6442	0.8791	1.0868	0.049*	
C7	0.67724 (15)	0.9214 (2)	0.96006 (13)	0.0382 (4)	
H7	0.6509	0.8949	0.8975	0.046*	
C8	0.77860 (15)	1.0250 (2)	0.97828 (12)	0.0357 (4)	
C9	0.82092 (16)	1.0873 (2)	1.06805 (13)	0.0431 (5)	
H9	0.7857	1.0592	1.1188	0.052*	
C10	0.91330 (17)	1.1886 (3)	1.08140 (14)	0.0485 (5)	
H10	0.9406	1.2280	1.1413	0.058*	
C11	0.96713 (16)	1.2336 (2)	1.00634 (14)	0.0466 (5)	
H11	1.0293	1.3036	1.0161	0.056*	
C12	0.92801 (15)	1.1742 (2)	0.91742 (13)	0.0396 (4)	
C13	0.83426 (15)	1.0682 (2)	0.90333 (12)	0.0347 (4)	
C14	0.73927 (19)	1.0981 (3)	0.74504 (15)	0.0540 (6)	
H14C	0.6614	1.1159	0.7566	0.081*	
H14B	0.7358	1.0488	0.6848	0.081*	
H14A	0.7796	1.1960	0.7454	0.081*	
C15	1.0673 (2)	1.3220 (3)	0.84993 (18)	0.0660 (7)	
H15C	1.0407	1.4174	0.8742	0.099*	
H15A	1.0896	1.3416	0.7899	0.099*	
H15B	1.1334	1.2829	0.8929	0.099*	

### Atomic displacement parameters (Å^2^)

**Table:**

	*U*^11^	*U*^22^	*U*^33^	*U*^12^	*U*^13^	*U*^23^
Cl1	0.0603 (3)	0.0672 (4)	0.0591 (4)	−0.0213 (3)	0.0158 (3)	0.0057 (3)
S1	0.0417 (3)	0.0445 (3)	0.0344 (3)	−0.00324 (19)	0.00221 (18)	0.0015 (2)
O1	0.0592 (8)	0.0645 (10)	0.0361 (8)	−0.0086 (7)	0.0025 (6)	0.0093 (7)
O2	0.0537 (7)	0.0392 (8)	0.0328 (7)	0.0012 (6)	0.0124 (6)	−0.0022 (6)
O3	0.0484 (8)	0.0632 (10)	0.0446 (8)	−0.0134 (7)	0.0174 (6)	0.0012 (7)
C1	0.0436 (10)	0.0360 (10)	0.0382 (10)	−0.0011 (8)	0.0122 (8)	0.0004 (8)
C2	0.0542 (11)	0.0497 (13)	0.0315 (9)	−0.0044 (9)	0.0074 (8)	−0.0008 (9)
C3	0.0438 (10)	0.0453 (12)	0.0374 (10)	−0.0059 (8)	0.0026 (8)	−0.0015 (9)
C4	0.0362 (9)	0.0339 (10)	0.0377 (10)	0.0025 (7)	0.0078 (7)	−0.0004 (8)
C5	0.0405 (9)	0.0382 (11)	0.0370 (10)	0.0060 (8)	0.0084 (8)	0.0046 (8)
C6	0.0443 (10)	0.0441 (11)	0.0343 (10)	−0.0009 (8)	0.0087 (8)	0.0013 (8)
C7	0.0438 (10)	0.0370 (11)	0.0349 (9)	0.0048 (8)	0.0101 (8)	0.0029 (8)
C8	0.0373 (9)	0.0365 (10)	0.0339 (9)	0.0061 (7)	0.0078 (7)	0.0024 (8)
C9	0.0453 (10)	0.0518 (13)	0.0333 (9)	0.0036 (9)	0.0100 (8)	0.0032 (9)
C10	0.0472 (11)	0.0616 (14)	0.0350 (10)	0.0001 (10)	0.0019 (8)	−0.0040 (9)
C11	0.0388 (10)	0.0536 (13)	0.0462 (12)	−0.0048 (8)	0.0042 (8)	−0.0020 (10)
C12	0.0374 (9)	0.0438 (12)	0.0394 (10)	0.0033 (8)	0.0116 (8)	0.0039 (9)
C13	0.0381 (9)	0.0337 (10)	0.0332 (9)	0.0049 (7)	0.0083 (7)	−0.0010 (8)
C14	0.0607 (13)	0.0573 (14)	0.0412 (11)	0.0048 (10)	0.0009 (9)	0.0042 (10)
C15	0.0583 (13)	0.0776 (18)	0.0665 (16)	−0.0211 (12)	0.0230 (11)	0.0056 (13)

### Geometric parameters (Å, °)

**Table:**

Cl1—C1	1.7173 (19)	C7—C8	1.461 (3)
S1—C1	1.7115 (19)	C7—H7	0.9300
S1—C4	1.7292 (18)	C8—C13	1.401 (2)
O1—C5	1.224 (2)	C8—C9	1.408 (3)
O2—C13	1.376 (2)	C9—C10	1.367 (3)
O2—C14	1.428 (2)	C9—H9	0.9300
O3—C12	1.371 (2)	C10—C11	1.394 (3)
O3—C15	1.425 (2)	C10—H10	0.9300
C1—C2	1.350 (3)	C11—C12	1.381 (3)
C2—C3	1.409 (3)	C11—H11	0.9300
C2—H2	0.9300	C12—C13	1.405 (3)
C3—C4	1.367 (2)	C14—H14C	0.9600
C3—H3	0.9300	C14—H14B	0.9600
C4—C5	1.467 (2)	C14—H14A	0.9600
C5—C6	1.474 (3)	C15—H15C	0.9600
C6—C7	1.336 (3)	C15—H15A	0.9600
C6—H6	0.9300	C15—H15B	0.9600
			
C1—S1—C4	90.57 (9)	C10—C9—C8	120.74 (18)
C13—O2—C14	115.51 (15)	C10—C9—H9	119.6
C12—O3—C15	117.37 (17)	C8—C9—H9	119.6
C2—C1—S1	113.76 (14)	C9—C10—C11	120.79 (18)
C2—C1—Cl1	126.07 (15)	C9—C10—H10	119.6
S1—C1—Cl1	120.16 (11)	C11—C10—H10	119.6
C1—C2—C3	111.04 (17)	C12—C11—C10	119.92 (19)
C1—C2—H2	124.5	C12—C11—H11	120.0
C3—C2—H2	124.5	C10—C11—H11	120.0
C4—C3—C2	113.79 (17)	O3—C12—C11	124.53 (17)
C4—C3—H3	123.1	O3—C12—C13	115.79 (16)
C2—C3—H3	123.1	C11—C12—C13	119.67 (17)
C3—C4—C5	131.18 (17)	O2—C13—C8	119.14 (16)
C3—C4—S1	110.84 (14)	O2—C13—C12	120.23 (16)
C5—C4—S1	117.97 (13)	C8—C13—C12	120.53 (17)
O1—C5—C4	119.86 (17)	O2—C14—H14C	109.5
O1—C5—C6	122.29 (18)	O2—C14—H14B	109.5
C4—C5—C6	117.85 (16)	H14C—C14—H14B	109.5
C7—C6—C5	121.68 (17)	O2—C14—H14A	109.5
C7—C6—H6	119.2	H14C—C14—H14A	109.5
C5—C6—H6	119.2	H14B—C14—H14A	109.5
C6—C7—C8	127.37 (18)	O3—C15—H15C	109.5
C6—C7—H7	116.3	O3—C15—H15A	109.5
C8—C7—H7	116.3	H15C—C15—H15A	109.5
C13—C8—C9	118.32 (17)	O3—C15—H15B	109.5
C13—C8—C7	119.08 (16)	H15C—C15—H15B	109.5
C9—C8—C7	122.57 (17)	H15A—C15—H15B	109.5
			
C4—S1—C1—C2	0.09 (17)	C13—C8—C9—C10	0.6 (3)
C4—S1—C1—Cl1	179.16 (12)	C7—C8—C9—C10	−177.48 (18)
S1—C1—C2—C3	−0.1 (2)	C8—C9—C10—C11	0.5 (3)
Cl1—C1—C2—C3	−179.14 (15)	C9—C10—C11—C12	−0.8 (3)
C1—C2—C3—C4	0.1 (3)	C15—O3—C12—C11	−3.8 (3)
C2—C3—C4—C5	−178.76 (19)	C15—O3—C12—C13	177.16 (18)
C2—C3—C4—S1	−0.1 (2)	C10—C11—C12—O3	−179.22 (18)
C1—S1—C4—C3	−0.02 (15)	C10—C11—C12—C13	−0.2 (3)
C1—S1—C4—C5	178.87 (15)	C14—O2—C13—C8	109.94 (19)
C3—C4—C5—O1	176.0 (2)	C14—O2—C13—C12	−73.6 (2)
S1—C4—C5—O1	−2.6 (2)	C9—C8—C13—O2	174.84 (15)
C3—C4—C5—C6	−4.6 (3)	C7—C8—C13—O2	−7.0 (2)
S1—C4—C5—C6	176.79 (13)	C9—C8—C13—C12	−1.6 (3)
O1—C5—C6—C7	−9.3 (3)	C7—C8—C13—C12	176.61 (16)
C4—C5—C6—C7	171.31 (17)	O3—C12—C13—O2	4.1 (3)
C5—C6—C7—C8	176.47 (17)	C11—C12—C13—O2	−175.02 (16)
C6—C7—C8—C13	175.42 (18)	O3—C12—C13—C8	−179.52 (16)
C6—C7—C8—C9	−6.5 (3)	C11—C12—C13—C8	1.4 (3)

### Hydrogen-bond geometry (Å, °)

**Table:**

*D*—H···*A*	*D*—H	H···*A*	*D*···*A*	*D*—H···*A*
C2—H2···O1^i^	0.93	2.53	3.209 (2)	130
C15—H15A···O2^ii^	0.96	2.55	3.423 (3)	151

Symmetry codes: (i) *x*, −*y*+3/2, *z*+1/2; (ii) −*x*+2, *y*+1/2, −*z*+3/2.
